# Uptake of Vaccinations among Children with Chronic Diseases Is Affected by Knowledge Gaps and Implementation Challenges in Italy

**DOI:** 10.3390/vaccines9111217

**Published:** 2021-10-20

**Authors:** Alessandra Beni, Sara Mazzilli, Elisabetta Bellino, Giorgio Costagliola, Elena Ferretti, Pier Luigi Lopalco, Lara Tavoschi, Diego Giampietro Peroni

**Affiliations:** 1 Department of Clinical and Experimental Medicine, Section of Pediatrics, University of Pisa, 56126 Pisa, Italy; alessandrabeni95@gmail.com (A.B.); elisabettabellino@libero.it (E.B.); giorgio.costagliola@hotmail.com (G.C.); e.ferretti@hotmail.it (E.F.); 2 Scuola Normale Superiore, 56126 Pisa, Italy; sara.mazzill@gmail.com; 3 Department of Translational Research and New Technologies in Medicine and Surgery, University of Pisa, Via San Zeno 35, 56124 Pisa, Italy; 4 Department of Science, Biological Technologies and Environmental, University of Salento, Piazza Tancredi, 7, 73100 Lecce, Italy; p.lopalco@regione.puglia.it

**Keywords:** immunization, child, pediatrician, knowledge, practice, vaccination attitude, chronic disease, vaccine, guidelines

## Abstract

(1) Background: Children with chronic medical conditions may be at increased risk for severe complications related to vaccine-preventable infections. Therefore, additional booster doses or supplementary vaccines are recommended, over and above the routine immunization schedule for healthy children. The aim of this study was to investigate attitude, knowledge, and practices toward additional vaccinations for children affected by chronic conditions among pediatricians and parents. (2) Methods: This study is based on two surveys: (i) a national cross-sectional survey, targeting pediatrician working in hospitals or in the primary health sector; (ii) a local cross-sectional survey, targeting parents of children with a previous diagnosis of chronic disease. (3) Results: Despite the fact that most of the health professionals and parents interviewed had an overall positive vaccine attitude, most pediatricians did not show an adequate knowledge of additional vaccinations for children affected by chronic diseases. Moreover, the coverage of additional recommended vaccinations in chronic pediatric patients was low. (4) Conclusions: This research highlighted important existing challenges hampering optimal vaccination coverage among pediatric chronic patients, including knowledge gaps on tailored vaccination schedules among pediatricians and organizational issues. The ongoing review of the Italian national immunization plan is a not-to-be-missed-opportunity to include evidence-based, detailed, and comprehensive recommendations on vaccinations for children affected by chronic conditions.

## 1. Introduction

Immunization is one of the most important and cost-effective strategies in public health. It is estimated that each year, worldwide, vaccines prevent up to three million deaths. However, the benefits of vaccines go beyond the avoided deaths and are also reported in terms of the disability-adjusted life years (DALYs) avoided or quality adjusted life years (QALYs) gained. Therefore, vaccination also provides significant savings by avoiding the health costs associated with treating diseases [1].

Even in high-income countries, the level of vaccination coverage is generally high and there are population groups with suboptimal vaccination rate [2]. Those include children and adolescents with chronic medical conditions that may be at increased risk for severe complications of vaccine-preventable infections, due to underlying chronic medical conditions and administered treatments. These individuals may also be more exposed to infections, due to the frequent access to healthcare services for both outpatient and inpatient regimens [3]. However, little is known about the level of vaccine uptake and acceptance among this specific group of patients, as data on uptake and hesitancy are provided for the pediatric population at large [4].

In order to maximize immunization efficacy, vaccination schedules differ according to the underlying medical conditions and the degrees of immunosuppression. Additional vaccine doses, more frequent booster doses, and supplementary vaccines could be indicated, in addition to the routine schedule used for healthy children [5].

National immunization schedules of several high-income countries include sections focused on immunization strategies for special groups, including chronic patients. However, the variety of possible conditions and heterogeneity of patients make the development of guidelines a difficult and challenging process. Immunization schedules of different countries differ not only for the medical conditions identified as risk groups, but also for vaccines recommended for each condition. Furthermore, scientific evidence behind these guidelines is often limited [3].

In the Italian National Immunization Plan (PNPV), immunization recommendations for chronic patients are provided; however, they are listed by vaccine, rather than by disease. This may constitute a barrier to easy consultation by pediatric specialists, especially considering that the medical conditions mentioned are often not described in detail. Therefore, it is up to pediatricians to identify additional resources (e.g., guidelines from scientific societies), in order to provide the best-suited immunization plan for their patients with chronic conditions [6]. As a consequence, pediatricians’ level of knowledge about specific vaccination recommendations for chronic patients may largely influence subsequent uptake.

The Italian national health-care system (NHS) is a regionally-based and organized into three levels: national, regional, and local. The national level is responsible for establishing the general objectives and fundamental principles of the NHS. The 19 regions and two autonomous provinces (R&AP) are responsible for organizing and delivering health care. Within each region, at the local level, local health authorities (LHA) deliver public health services, community health services and primary care directly, and secondary and specialist care directly or through either public hospitals or accredited private providers [7]. In this scenario, through the PNPV, the Ministry of Health has taken the lead in health-care prevention and vaccination planning. Then, the R&AP are in charge of organizing and implementing the current vaccination strategy at the local level, based on PNPV provisions. However, each region has the possibility to expand PNPV prescriptions, based on regional needs or specific epidemiological context. So, at least in some regions, including Tuscany, regional immunization plans are available [8].

This study had two main aims: (i) to explore attitude, knowledge, and practices toward the additional vaccinations recommended for children affected by chronic conditions, in a sample of Italian pediatricians and among parents of children affected by chronic conditions; (ii) to investigate vaccination history and the additional vaccination performed in a sample of children affected by chronic conditions.

## 2. Materials and Methods

This study is based on two surveys: (i) a national, cross-sectional survey targeting pediatrician working in hospitals or the primary health sector (family pediatricians); (ii) a local, cross-sectional survey targeting parents of children from 6 months to 18 years old with a previous diagnosis of chronic or onco-hematological disease.

### 2.1. Survey Targeting Pediatricians

#### Survey Design

The survey was developed electronically on a Microsoft^®^ form by the research team. The questionnaire was piloted, and subsequently revised, according to the remarks of the participating pediatricians. The link to the final version of the survey was sent, via email, to the Italian Society of Preventive and Social Pediatrics (SIPPS) members, as well as to pediatricians within the Pediatric Network of the North-West Tuscany LHA. The email included a detailed explanation, regarding the purpose and the non-compulsory nature of the study and clarified that respondents’ anonymity was guaranteed. Respondents had to complete the informed consent form before proceeding to the survey. The survey was conducted from March to June 2021.

The self-administered, anonymous survey (Figure S1) consisted of four parts, investigating the following areas: (i) personal information of responders (age, sex, geographical area of service, and sub-specialization); (ii) vaccine attitude and vaccination promotion practices implemented by pediatrician towards the pediatric population not affected and affected by chronic diseases; (iii) knowledge on additional vaccinations recommended for patients suffering from chronic diseases; (iv) pediatricians’ perception of their knowledge on additional vaccinations recommended for chronic patients, willingness to play a role in recommending the necessary vaccinations for these patients, and suggestions on how they would prefer to be informed on the subject. The survey included both categorical and five-point Likert scale questions (2 levels of agreement, 1 neutral choice, and 2 levels of disagreement).

Knowledge on the vaccinations recommended for chronic patients was considered adequate if respondents indicated as additional vaccination: (i) the ones mentioned in the Italian PNPV, (ii) vaccination mentioned in the Italian PNPV and in other countries immunization plans. On the contrary, knowledge was considered inadequate if respondents: (i) did not indicate all the additional vaccinations mentioned in the Italian PNPV, (ii) indicated vaccinations not mentioned in Italian or other countries immunization plans or indicated vaccination that was contraindicated for the chronic diseases considered.

### 2.2. Survey Targeting Parents of Children with a Previous Diagnosis of Chronic or Onco-Hematological Disease

#### 2.2.1. Study Setting

Tuscany is a central Italian region of about 3.7 million inhabitants [9]. The regional healthcare service is organized into three LHAs: North-West Tuscany LHA, South-East Tuscany LHA, and Central Tuscany LHA. The Azienda Ospedaliero-Universitaria Pisana (AOUP) is a large, teaching, tertiary hospital and represents the referral hospital for the North-West Tuscany LHA [10].

#### 2.2.2. Survey Design

The survey was developed by the research team. The questionnaire was piloted and revised according to the feedback of the participating subjects. From November 2019 to February 2020, the parents whose children attended outpatient visits in the pediatric clinic of AOUP were recruited during routine follow up visits and the survey was administered face-to-face by an operator. Parents were asked to sign the informed consent that clarified the non-compulsory and anonymous nature of the survey. In May 2020, due to restrictions for the COVID-19 pandemic, the recruitment was completed through telephone interview. Eligibility criteria included: (i) having a child, from 6 months to 18 years old, with a previous diagnosis of chronic or onco-hematological disease; (ii) the chronic or onco-hematological disease had to be listed in the PNPV or in the immunization schedule of Tuscany region as at major risk for infections and, therefore, additional vaccinations were recommended to children with those conditions (in Table S1 details on the chronic or onco-hematological disease considered).

The survey (Figure S2) investigated three main areas: (i) attitudes toward vaccination and the child’s vaccination history; (ii) source of information on vaccinations; (iii) received recommendation and additional vaccinations performed to children affected by chronic conditions. The survey included both categorical and open-ended questions.

### 2.3. Children Immunization Status According the Immunization Information System

The immunization status of the children with a previous diagnosis of chronic or onco-hematological disease of interviewed parents were gathered through the Tuscany immunization information system (SISPC). Children immunization status was considered up-to-date if they had received all the routine vaccinations scheduled for their age in the PNPV.

### 2.4. Data Analysis

We conducted a descriptive analysis of the main sample characteristics. The descriptive summaries are provided as a percentage of the distribution of the level variables.

### 2.5. Ethical Approval

The study was approved by the ethics committee of the University of Pisa in two separate protocols, one for each survey. The number of protocols of the survey targeting pediatricians is: 0064597/2021; the number of protocols of the survey that targeted parents is 0113186/2019.

## 3. Results

### 3.1. Respondents to the Survey Targeting Pediatricians

#### 3.1.1. Sample Characteristics

A total of 238 pediatricians completed the survey, of which 138 (58.0%) were female. Of the respondents, 46 (19.3%) worked in a hospital or specialist clinics, 172 (72.3%) worked as family pediatricians, and 20/238 (8.4%) were self-employed. Table 1 shows detailed sample characteristics.

#### 3.1.2. Vaccine Attitude of Pediatricians

The interviewed pediatricians showed a positive attitude for both routine vaccinations in the healthy population and additional vaccinations in children with chronic conditions. Indeed, only a pediatrician disagreed with the sentence: “I believe that the vaccinations recommended in the Italian PNPV are important for all children”. All the respondents strongly agreed (217–91.2%) or agreed (21–8.8%) with the sentence “I believe that additional vaccinations for patients at risk are important”.

#### 3.1.3. Practices towards Vaccination

Among the 217 respondents working in a hospital, in specialist clinics, or as family pediatrician, 212 (97.7%) regularly registered information on the children vaccination status during visits, while 5 pediatricians (2.3%) recorded vaccination history only at the first visit. Among those pediatricians regularly registering information on children’s vaccination status, 116 (55.8%) asked for written or online vaccination certificates, while 92 (44.2%) asked for parents’ oral reports. Most of the pediatricians (197, 90.8%) dedicated time to discuss the importance of vaccinations with the parents of all their patients, 7 (3.2%) dedicated time to discuss the importance of vaccinations with only the parents of children affected by chronic diseases, and 13 (6.0%) did not always find the time. Vaccination were mainly recommended orally (148, 68.2%), while only 29.0% (69) of pediatricians provided written prescriptions. Most of respondents recommend always (158, 72.8%) or often (47, 21.7%) considering additional vaccination for children affected by chronic diseases, 10 (4.6%) recommended additional vaccination occasionally, and 2 (0.9%) did not give additional recommendations. While 66 (30.4%) of the pediatricians administered vaccinations to their non-chronic patients, 82 (37.8%) administered vaccinations to children affected by chronic diseases. Table 2 shows answers stratified by working place.

#### 3.1.4. Perceived Knowledge about Vaccinations for Children with Chronic Diseases

While the 27.7% (65/238) of respondents felt they had sufficient knowledge about the additional vaccinations recommended in patients with primary or secondary immunity deficiency, the 60.9% (145/238) of them felt they had sufficient knowledge about the additional vaccinations recommended in patients with other chronic diseases.

#### 3.1.5. Knowledge about Vaccination for Children with Chronic Diseases

As shown in Table 3, the majority of pediatricians have inadequate knowledge, regarding vaccination recommended in children with chronic diseases, with a minimum of 0.5% (1/237) of pediatricians with an adequate knowledge of vaccination recommended in patients with severe chronic kidney disease and a maximum of 17.3% (41/237) of pediatricians with an adequate knowledge of vaccination recommended in cancer patients in the maintenance phase.

### 3.2. Respondents to the Survey Targeting Parents

#### 3.2.1. Sample Characteristics

A total of 103 parents were recruited, of which 32 had only one child, and 71 had more than one child. A total of 20 had a child affected by two or more chronic diseases. In the case of multiple diagnosis of chronic conditions, the answers given, in regard to recommendations for additional vaccines, were included in the analysis of each condition. Table S2 shows the total number of surveys collected by medical condition.

#### 3.2.2. Parents’ Vaccination Attitude and Children Vaccination History

Parents interviewed showed a positive attitude for both routine vaccinations and additional vaccinations recommended in children with chronic conditions. Over 71 respondents reporting to have more than one child 68/71 (98.5%) administered to their child/children not affected by chronic conditions all the immunization recommended by the pediatricians. While 85.4% of parents (88/103) reported that their children, affected by chronic diseases, received all the routine vaccinations. Among the 15 parents who reported that their child, affected by chronic conditions, did not receive all the routine vaccination, 6/103 (5.8%) reported to have interrupted the immunization of their children because of fear of adverse reactions or doubts on the effectiveness of vaccines, and 9/103 (8.7%) reported that their children had not received all the routine vaccinations, due to their chronic disease. When asked if vaccinations could improve the health of children with chronic diseases, the majority of parents (88/103, 85.4%) responded yes, 11/103 (10.7%) partially, 2/103 (1.9%) responded no, and 2/103 (1.9%) preferred not to answer.

#### 3.2.3. Source of Information on Vaccinations

Most of the respondents (96/103 (93.2%)) reported to have acquired information on vaccine from pediatricians or medical doctor at the vaccination services. Only 4/103 (3.9%) reported as main source of information other family members and 3/103 (2.9%) declared they collected information on vaccines on their own initiative.

#### 3.2.4. Received Recommendation and Additional Vaccinations Performed to Children Affected by Chronic Conditions

About half of the respondents (54/103 (52.4%)) received at least one recommendation to administer additional vaccinations to their child who is affected by a chronic condition; of those, 36/54 (66.7%) received only verbal recommendation, 14/54 (25.9%) received written recommendation, 1/54 (1.9%) directly received the appointment for the additional vaccine, while 3/54 (5.6%) did not remember how they were informed about the additional vaccination. Out of the 54 parents who received the recommendation for the additional vaccination, 32/54 (59.3%) administered all the additional vaccinations recommended to their child, 3/54 (5.6%) administered just some of them, 2/54 (3.7%) had the vaccine scheduled, 16/54 (29.6%) did not administer any of the recommended vaccinations to their child, and one parent (1.9%) did not remember if his child received additional vaccinations. The additional vaccinations administered were reported to the pediatric specialist by 21/35 (60%) parents, of which 17/21 (80.95%) gave only verbal feedback, 3/21 (14.3%) showed the immunization certificate of vaccination, and 1/21 (4.8%) sent an email.

Table 4 shows the percentage of additional vaccinations recommended and those actually received for each medical condition.

### 3.3. Children Immunization Status According the Immunization Information System

It was possible to check the immunization status on SISPC for 72 out of 103 child of the respondents interviewed. A total of 32 respondents were not residents of the Tuscany region; therefore, the immunization status of their children was not available on SISPC. Out of the 57/72 (79%) parents who reported to have administered all the recommended routine immunization to their children, only 37/52 (71.2%) actually had all the routine vaccinations reported for their children.

## 4. Discussion

Children and adolescents with chronic medical conditions may be at increased risk for severe complications related to vaccine-preventable infections. Therefore, additional booster doses or supplementary vaccines are recommended over and above routine immunization schedule for healthy children. To the best of our knowledge this is the first Italian study investigating attitude, knowledge and practices toward additional vaccinations for children affected by chronic condition among pediatricians and parents.

The majority of health professionals and parents interviewed had an overall positive attitude towards vaccinations. These results are in line with what was previously reported in the literature both for pediatricians [11] and for parents [4]. However, with regard to the parents, results suggest they have a higher vaccine acceptance when vaccination regards healthy children (98.5%), as compared to children suffering from chronic diseases (85.4%). Therefore, specialist and family pediatricians should dedicate additional time to advise on and discuss vaccinations with parents of child affected by chronic diseases answering their questions and possible concerns.

In our study, the majority of pediatricians had an inadequate knowledge about vaccination recommended for patients affected by chronic conditions. Esposito and colleagues [12] in a previous study already suggested that low influenza vaccination coverage among children with high risk medical conditions could be due to poor knowledge among pediatricians. However, our findings show that most pediatricians also do not have adequate knowledge of other additional vaccinations for children affected by chronic diseases (i.e, pneumococcal polysaccharide 23-valent additional vaccine or chickenpox booster dose). Furthermore, a number of pediatricians in our study indicates live and attenuated vaccines, as vaccinations or additional doses adequate for patients with onco-hematological disease, although these vaccines are contraindicated in these patients. The knowledge gap on this topic is also confirmed by the pediatricians themselves, indeed more than 40% of them do not felt they had sufficient knowledge about the additional vaccinations recommended in patients with chronic diseases.

Reasons for poor knowledge could be the absence of dedicated training in vaccinology in university curricula, as well as the lack of comprehensive documentation to provide all the recommendations for immunization in children with chronic diseases.

In agreement with the literature [4], our results confirm that healthcare workers are considered the preferred source of information on vaccination by parents. Moreover, Pandolfi and colleagues [13] have shown that receiving a specific recommendation for influenza immunization by any physician is a single strong determinant of influenza immunization uptake in children with chronic diseases. This opportunity to contrast vaccine hesitancy is, however, hampered by the reported lack of specific knowledge when it comes to vaccination schedules adapted to chronic patients’ needs. For this reason, it is fundamental to provide pediatricians with all the tools available to address any knowledge gaps and to empower them to recommend comprehensive immunization schedule to meet the needs of their fragile patients.

In addition, organizational issues could cause sub-optimal immunization coverage. According to our findings, recommendations on additional vaccinations and referral were mainly given by specialists to parents orally. Yet, written prescriptions and digital reminder systems should be preferred because they are more effective communication methods as suggested by the existing evidence [14,15]. In our study, an overall low adherence to the recommended vaccinations was observed, with only 26.5% of children with chronic disease received influenza vaccine, while, in France, where the National Health Insurance sends free vaccination voucher at home, the vaccination rate reached 46.5% [16]. Also, feedbacks to specialist pediatricians about administered vaccinations were mostly left to verbal reports by parents. This approach may be subject to a number of errors, such as lack of timeliness, lack of reporting or inaccurate feedback. The occurrence of the latter was shown in our study by the difference in vaccination coverage when this information was reported by the parents or extrapolated from the regional vaccination information system. For this reason, access to vaccination registry among specialist in hospital settings would be desirable in order to minimize missed opportunities to vaccinate.

In addition, as children with chronic disease are frequently hospitalized, it can be challenging for them to attend community appointments for vaccination. In our sample, less than 40% of pediatricians directly administer vaccinations to their chronic patients. Napolitano et al. [17] have already underlined the need to take advantage of medical visits and hospitalization to actively offer vaccination, including routine vaccinations for this patients. Previous studies [18,19] demonstrated that influenza immunization in pediatric tertiary care hospital is a feasible and acceptable way to reach children with chronic diseases, and it is also an effective approach to reduce missed opportunities. Ensuring continuous interaction between specialists and community vaccination services is, therefore, essential to contribute to improving vaccination coverage among chronic pediatric patients. This study had some limitations. First, a limited number of pediatricians responded to the survey. However, it was not possible to estimate the response rate, due to the dissemination approach chosen. It is possible that pediatricians with a positive vaccination attitude may be overrepresented in our study sample, as an effect of the convenience sampling approach applied. The survey to parents was performed in a single centre and has limited geographical representativeness. Data collection was made through face-to-face interview and this may have led to social desirability and recall bias. Despite these limitations, findings from this survey contribute to underlying gaps in the knowledge of pediatricians and the low coverage regarding recommended vaccinations in patients with chronic diseases.

## 5. Conclusions

Our findings highlighted important existing challenges hampering optimal vaccination coverage among pediatric chronic patients, including knowledge gaps on tailored vaccination schedules among pediatricians and organizational issues, such as referral systems and continuity of care between hospital and community vaccination services.

The ongoing review of the Italian PNPV is a not-to-be-missed opportunity to include evidence-based, detailed, and comprehensive recommendations on vaccinations for children affected by chronic conditions to guide pediatricians and contribute to contrast vaccine hesitancy. We advocate for the development of an operational guidelines to foster implementation of: (i) standard patient-centered procedure for the recommendation and administration of vaccinations, promoting harmonization and close collaboration between specialist pediatricians and vaccination services, and (ii) an information vaccination system accessible by all the health care workers involved in the process.

## Figures and Tables

**Table 1:** Respondent characteristics.

Variable		N (%)
Gender	Male	100 (42.0%)
	Female	138 (58.0%)
Age	30–40	8 (3.4%)
	40–50	15 (6.3%)
	>50	215 (90.3%)
Working settings	Family pediatricians	172 (72.3%)
	Pediatricians working in a hospital	42 (17.6%)
	Pediatricians working in specialist clinics	4 (1.7%)
	Pediatricians working as self-employed	20 (8.4%)
Geographical Area	North	83 (34.9%)
	Centre	81 (34.0%)
	South	63 (26.5%)
	Islands	11 (4.6%)

**Table 2:** Vaccination practices, according the working place of respondents.

Question	Answers	Family Pediatricians	Pediatrician Working in Hospital	Pediatrician Working in Specialist Clinics
		N 172	N 41	N 4
Do you request information about the vaccination status?	Yes, through paper or online vaccination certificates	61 (34.5%)	31 (75.6%)	1 (25.0%)
	Yes, through parents’ oral reports	110 (63.9%)	6 (14.6%)	3 (75.0%)
	No, only at the 1st visit	1 (0.6%)	4 (9.7%)	-
	No	-	-	-
Do you spend time discussing the importance of vaccinations with parents?	Yes, for all my patients	168 (97.7%)	25 (61%)	4 (100%)
	Yes, only for children with chronic diseases	1 (0.6%)	6 (14.6%)	-
	No, I do not always find the time	3 (1.7%)	10 (24.4%)	-
	No	-	-	-
Type of vaccine recommendation	Oral	118 (68.6%)	27 (65.9%)	3 (75.0%)
	Medical prescription	54 (31.4%)	14 (34.1%)	1 (25.0%)
Do you recommend additional vaccination for children affected by chronic diseases?	Always	131 (76.2%)	24 (58.5%)	3 (75.0%)
	Often	31 (18.0%)	15 (36.6%)	1 (25.0%)
	Occasionally	8 (5.7%)	2 (4.9%)	-
	Never	2 (1.2%)	-	-
Do you administer vaccinations to your patients?	Always/Often	60 (34.9%)	4 (9.8%)	2 (50.0%)
	Occasionally/Never	112 (65.1%)	37 (90.2%)	2 (50.0%)
Do you administer vaccinations to children affected by chronic diseases?	Always/Often	75 (43.6%)	4 (9.8%)	3 (75.0%)
	Occasionally/Never	97 (56.4%)	37 (90.2%)	1 (25.0%)

**Table 3:** Percentage of respondents with an adequate or inadequate knowledge about vaccination recommended in children with chronic diseases.

Diseases	Respondents with Adequate Knowledge	Respondents with Inadequate Knowledge
Bronchial asthma in chronic treatment	17 (7.1%)	221 (92.9%)
Diabetes mellitus	7 (2.9%)	231 (98.1%)
Severe obesity	30 (12.6%)	208 (87.4%)
Chronic heart disease	15 (6.3%)	223 (93.7%)
Conditions at increased risk of aspiration of secretions	21 (8.8%)	217 (91.2%)
Cochlear implant	20 (8.4%)	218 (91.6%)
Sickle Cell Anemia or Major Thalassemia with surgical or functional asplenia	10 (4.2%)	228 (95.8%)
Severe chronic kidney disease	1 (0.5%)	237 (99.5%)
Cancer patients in the maintenance phase	41 (17.3%)	197 (82.7%)

**Table 4:** Percentage of additional vaccinations recommended and actually administered by medical condition. In case a child was diagnosed with more than one chronic condition, responses were included in the analysis of each condition.

Medical Condition	IIV *		PPV23 °		HAV §	
	Recommended	Received	Recommended	Received	Recommended	Received
Chronic respiratory or lung disease	13/20 (65%)	8/20 (40%)	2/20 (10%)	1/20 (5%)	-	-
Chronic heart disease	4/6 (67%)	1/6 (17%)	0/6 (0%)	0/6 (0%)	-	-
Conditions at increased risk of aspiration of secretions	6/13 (46%)	4/13 (31%)	-	-	-	-
Adrenal cortical insufficiency	0/4 (0%)	1/4 (25%)	-	-	-	-
Severe chronic kidney disease	-	-	0/4 (0%)	0/4 (0%)	-	-
Chronic liver disease	0/1 (0%)	0/1 (0%)	0/1 (0%)	0/1 (0%)	0/1 (0%)	0/1 (0%)
Diabetes mellitus	9/13 (69%)	4/13 (31%)	0/13 (0%)	0/13 (0%)	-	-
Haemophilia and other coagulation disorders	1/4 (25%)	1/4 (25%)	0/4 (0%)	0/4 (0%)	0/4 (0%)	0/4 (0%)
Anemia and hemoglobinopathy	6/12 (50%)	4/12 (33%)	-	-	-	-
Severe obesity	1/3 (33%)	1/3 (33%)	-	-	-	-
Chronic inflammatory diseases and malabsorption syndromes	1/2 (50%)	1/2 (50%)	-	-	-	-
Patients with cochlear implant	-	-	5/6 (83%)	5/6 (83%)	-	-
Patients with cerebrospinal fluid fistula	1/1 (100%)	1/1 (100%)	0/1 (0%)	0/1 (0%)	-	-
Patient waiting for major surgery	0/2 (0%)	0/2 (0%)	-	-	-	-
Primary or secondary immunodeficiencies	5/28 (18%)	2/28 (7%)	0/28 (0%)	0/28 (0%)	-	-
Solid organ transplantation	0/1 (0%)	0/1 (0%)	0/1 (0%)	0/1 (0%)	-	-
Surgical or functional asplenia	5/7 (71%)	3/7 (43%)	0/7 (0%)	0/7 (0%)	-	-
Total	52/117 (44%)	31/117 (27%)	7/91 (8%)	6/91 (7%)	0/5 (0%)	0/5 (0%)

* IIV = Inactivated influenza vaccine; ° PPV23 = Pneumococcal polysaccharide 23- valent vaccine; § HAV = Hepatitis A vaccine. In this table are reported vaccines that are administered only as additional vaccination in child with chronic condition and not included in the routine immunization schedule. Therefore, Meningococcal B, Meningococcal C, and Chickenpox vaccines that are recommended both at any age in some chronic diseases and, since 2017, have been included in the routine immunization schedule, are not reported in this table.

## Data Availability

Data used in the current study, data dictionaries and study protocol will be available immediately and ending 36 months following publication. Data will be available to anyone who wishes to access the data. To request access to the data, please write to sara.mazzilli@sns.it.

## Supplementary Materials

The following are available online at https://www.mdpi.com/article/10.3390/vaccines9111217/s1, Table S1: List of the chronic or onco-haematological diseases considered in the eligibility criteria, Table S2: Total number of surveys collected by medical condition, Figure S1: Survey targeting pediatricians: “Immunization in children with chronic diseases”, Figure S2: Survey targeting Parents of Children with a Diagnosis of Chronic Disease.
