# Supplementary material for: Uptake of Vaccinations among Children with Chronic Diseases Is Affected by Knowledge Gaps and Implementation Challenges in Italy
Source: Vaccines (Basel). 2021 Oct 20;9(11):1217. doi: 10.3390/vaccines9111217 (PMC8623218; doi:10.3390/vaccines9111217)
Supplement: Supplementary file 1 [file vaccines-09-01217-s001.zip › Figure S1.pdf]

Figure S1

Survey targeting pediatricians: “Immunization in children with chronic diseases”.

1. Age:

- <30
- 31–40
- 41–50
- >50

2. Gender

- M
- F

3. In which region do you work?
4. In which province do you work?
5. Qualification

- Resident doctor in pediatric
- Specialist doctor working in hospital
- Primary care pediatrician
- Others:

6. In which field are you most interested?

- Onco haematology
- Endocrinology
- Neurology
- Pneumology/Allergology
- Immunology/Rheumatology
- Gastroenterology
- Nephrology
- Infectious diseases
- Cardiology
- General pediatrics
- Others:

7. “I believe that vaccinations recommended in the Italian PNPV are important for all children”.

- Strongly agree
- Agree
- Neutral
- Disagree
- Strongly disagree

8. “I believe that additional vaccinations for patients at risk are important”

- Strongly agree
- Agree
- Neutral
- Disagree
- Strongly disagree

9. Do you ask about the vaccination status of your patient during routine visit?

- Yes, through parents’ oral reports
- Yes, through paper or online vaccination certificates
- No, only at the first visit

- No, I did not ask about vaccination status of patients

10. Do you spend time discussing the importance of vaccination with parents during your clinical practice?

- Yes, for all of my patients
- Yes, but only for children with chronic diseases
- No, I do not always find time
- No, I never do it
- Others:

11. How do you recommend vaccinations to patients?

- Verbal recommendation
- Written recommendation
- Written recommendation in Treatment plan
- Others:

12. Do you administer routine vaccinations to all of your patients in your clinic?

- Always
- Often
- Occasionally
- Never

13. Do you recommend additional vaccinations for children suffering from chronic diseases?

- Always
- Often
- Occasionally
- Never

14. Do you administer additional vaccinations to children with chronic diseases in your clinic?

- Always
- Often
- Occasionally
- Never

15. In which medical conditions would you consider to postpone routine immunization schedule?

- Patients with suspect of primary immunodeficiency or patients with acquired immunodeficiency (e.g., solid organ or haematopoietic stem cell transplant, cancer chemotherapy or immunosuppressive therapies)
- Patients with other chronic diseases (asthma, obesity, chronic hepatitis, asplenia, chronic renal diseases, chronic heart disease...)
- Egg allergy (suspected or diagnosed), without history of anaphylaxis
- Premature newborns
- All of these conditions
- None of them

16. Which vaccinations must be postponed in a patient who needs to start immediately a high-level immunosuppressive therapy or in a patient who is getting diagnosed with a primary immunodeficiency?

- Pneumococcal conjugate 7- or 13- valent
- Measles-mumps-rubella + chickenpox
- Meningococcal B vaccine and Meningococcal C vaccine

- Inactivated influenza vaccine
- Diphtheria-Tetanus-Pertussis-Polio-Haemophilus influenza type B-Hepatitis B vaccine
- Rotavirus vaccine
- None of these vaccinations
- All of them

Answer with the additional vaccines (supplementary vaccines, additional doses or most frequent booster doses than routine immunization schedule proposed in PNPV) which you would recommend for each risk condition:

17. Bronchial asthma in chronic treatment:

- Pneumococcal conjugate 13- valent + pneumococcal polysaccharide 23- valent vaccines
- Chickenpox vaccine (if not immunized by natural infection)
- Quadrivalent meningococcal vaccine (ACWY)
- Meningococcal B vaccine
- Inactivated influenza vaccine
- Haemophilus influenza type B vaccine
- Hepatitis A vaccine
- All of them
- None of them

18. Diabetes mellitus:

- Pneumococcal conjugate 13- valent + pneumococcal polysaccharide 23- valent vaccines
- Chickenpox vaccine (if not immunized by natural infection)
- Quadrivalent meningococcal vaccine (ACWY)
- Meningococcal B vaccine
- Inactivated influenza vaccine
- Haemophilus influenza type B vaccine
- Hepatitis A vaccine
- All of them
- None of them

19. Severe Obesity:

- Pneumococcal conjugate 13- valent + pneumococcal polysaccharide 23- valent vaccines
- Chickenpox vaccine (if not immunized by natural infection)
- Quadrivalent meningococcal vaccine (ACWY)
- Meningococcal B vaccine
- Inactivated influenza vaccine
- Haemophilus influenza type B vaccine
- Hepatitis A vaccine
- All of them
- None of them

20. Chronic heart disease:

- Pneumococcal conjugate 13- valent + pneumococcal polysaccharide 23- valent vaccines
- Chickenpox vaccine (if not immunized by natural infection)
- Quadrivalent meningococcal vaccine (ACWY)
- Meningococcal B vaccine
- Inactivated influenza vaccine
- Haemophilus influenza type B vaccine
- Hepatitis A vaccine
- All of them

- None of them

21. Cochlear implant:
    - Pneumococcal conjugate 13- valent + pneumococcal polysaccharide 23- valent vaccines
    - Chickenpox vaccine (if not immunized by natural infection)
    - Quadrivalent meningococcal vaccine (ACWY)
    - Meningococcal B vaccine
    - Inactivated influenza vaccine
    - Haemophilus influenza type B vaccine
    - Hepatitis A vaccine
    - All of them
    - None of them

22. Conditions at increased risk of inhalation of secretions (e.g., ICP, neuromuscular diseases, other severe disabilities):
    - Pneumococcal conjugate 13- valent + pneumococcal polysaccharide 23- valent vaccines
    - Chickenpox vaccine (if not immunized by natural infection)
    - Quadrivalent meningococcal vaccine (ACWY)
    - Meningococcal B vaccine
    - Inactivated influenza vaccine
    - Haemophilus influenza type B vaccine
    - Hepatitis A vaccine
    - All of them
    - None of them

23. Sickle Cell Anemia or Major Thalassemia with surgical or functional asplenia:
    - Pneumococcal conjugate 13- valent + pneumococcal polysaccharide 23- valent vaccines
    - Chickenpox vaccine (if not immunized by natural infection)
    - Quadrivalent meningococcal vaccine (ACWY)
    - Meningococcal B vaccine
    - Inactivated influenza vaccine
    - Haemophilus influenza type B vaccine
    - Hepatitis A vaccine
    - All of them
    - None of them

24. Severe chronic kidney disease (nephrotic syndrome, dialysis...):
    - Pneumococcal conjugate 13- valent + pneumococcal polysaccharide 23- valent vaccines
    - Chickenpox vaccine (if not immunized by natural infection)
    - Quadrivalent meningococcal vaccine (ACWY)
    - Meningococcal B vaccine
    - Inactivated influenza vaccine
    - Haemophilus influenza type B vaccine
    - Hepatitis A vaccine
    - All of them
    - None of them

25. Patient with cancer receiving maintenance chemotherapy:
    - Pneumococcal conjugate 13- valent + pneumococcal polysaccharide 23- valent vaccines
    - Chickenpox vaccine (if not immunized by natural infection)
    - Quadrivalent meningococcal vaccine (ACWY)
    - Meningococcal B vaccine

- Inactivated influenza vaccine
- Haemophilus influenza type B vaccine
- Hepatitis A vaccine
- All of them
- None of them

26. Concerning immunization in patients with primary immunodeficiency or patients with acquired immunodeficiency (e.g., solid organ or haematopoietic stem cell transplant, cancer chemotherapy, or immunosuppressive therapies):

- I feel I have NOT ENOUGH information on additional recommended vaccines
- I feel I have ENOUGH information on additional recommended vaccines

27. Concerning immunization in patients with primary immunodeficiency or patients with acquired immunodeficiency (e.g., solid organ or hematopoietic stem cell transplant, cancer chemotherapy or immunosuppressive therapies):

- I think primary care pediatrician should inform the patient about recommended vaccinations
- I think specialist pediatrician should inform the patient about recommended vaccinations
- I think both primary care and specialist pediatrician should cooperate to recommend additional vaccinations
- I think MD working at the vaccination centres should give recommendations about immunization if pediatricians request them
- Others:

28. Concerning immunization in patients with other chronic diseases (asthma, obesity, chronic hepatitis, asplenia, chronic renal diseases, chronic heart disease...)

- I feel I have NOT ENOUGH information on additional recommended vaccines
- I feel I have ENOUGH information on additional recommended vaccines

29. Concerning immunization in patients with other chronic diseases (asthma, obesity, chronic hepatitis, asplenia, chronic renal diseases, chronic heart disease...)

- I think primary care pediatrician should inform the patient about recommended vaccinations
- I think specialist pediatrician should inform the patient about recommended vaccinations
- I think both primary care and specialist pediatrician should cooperate to recommend additional vaccinations
- I think MD working at the vaccination centres should give recommendations about immunization if pediatricians request them
- Others:

30. Concerning immunization in patients with other chronic diseases (asthma, obesity, chronic hepatitis, asplenia, chronic renal diseases, chronic heart disease...) I think PNPV include complete and easily accessible recommendations:

- Strongly agree

- Agree
- Neutral
- Disagree
- Strongly disagree

31. I am interested in receiving more information about additional vaccinations recommended in patients with chronic diseases:

- Strongly agree
- Agree
- Neutral
- Disagree
- Strongly disagree

32. How would you like to be informed about vaccinations recommended in patients with chronic diseases?

- Immunization schedule specific for each disease
- Conferences
- Webinar
- Mailing list
- Scientific literature
- Others:
