# Supplementary material for: Uptake of Vaccinations among Children with Chronic Diseases Is Affected by Knowledge Gaps and Implementation Challenges in Italy
Source: Vaccines (Basel). 2021 Oct 20;9(11):1217. doi: 10.3390/vaccines9111217 (PMC8623218; doi:10.3390/vaccines9111217)
Supplement: Supplementary file 1 [file vaccines-09-01217-s001.zip › Figure S2.pdf]

Figure S2

Survey targeting Parents of Children with a Diagnosis of Chronic Disease

1. Are you parent of a child suffering from chronic disease?

- Yes
- No

If "Yes", which disease/s are your child suffering from?

2. Are you parent of other children?

- Yes
- No

If yes, are they fully vaccinated according to the routine immunization schedule?

- Yes
- No
- Partially

If "No" or "Partially", why did you not follow recommendations of immunization in children?

- Fear of adverse events
- Doubt about the effectiveness of vaccines
- Contraindications
- Others, explain:

3. Which was your main source of information about vaccines?

- Family
- Friends
- TV
- Internet
- Social Networks
- Newspapers or online magazines
- Primary care doctor
- Health institutions (e.g., vaccination centres)
- Others:

4. Do you think that vaccination can improve the health of children suffering from chronic diseases?

- Yes
- No
- Partially

If "No" or "Partially", why?

5. Is your child fully vaccinated according to the routine immunization schedule proposed in Tuscany?

- Yes
- No
- Partially

If "No" or "Partially", why?

- Fear of adverse events
- Doubt about the effectiveness of vaccines
- Contraindications
- Others, explain:

6. As a child with chronic disease, your child is at increased risk for severe complications related to vaccine-preventable infections and should receive additional vaccines: are you aware about this information?

- Yes
- No

If "Yes", do you know which additional vaccines are recommended for your child?

7. How did you find out this recommendation?

- Recommendation given by Primary care paediatrician
- Recommendation given by Specialist paediatrician
- Recommendation given by vaccination service physician
- Individual research
- Others, explain:

8. If recommended, what method of communication has been used to give you information about additional vaccines?

- Oral recommendation
- Written medical prescription
- Written prescription in Treatment plan

9. Which additional vaccines did your child receive?

10. In which setting did your child receive the additional vaccines?

- Primary care clinic
- Vaccination centre
- Others, explain:

11. Did you update specialist doctor on additional vaccines administered?

- Yes
- No

If "Yes", which method of communication did you use?

- Oral communication
- Vaccination certificate
- Others, explain:.

12. If you did not get your child immunized with additional vaccines, which was the reason/s of your choice?

- Fear of adverse events
- Doubt about the effectiveness of vaccines
- Contraindications
- Difficulties in getting the additional vaccines
- Others, explain:
