# Supplementary material for: Uptake of Vaccinations among Children with Chronic Diseases Is Affected by Knowledge Gaps and Implementation Challenges in Italy
Source: Vaccines (Basel). 2021 Oct 20;9(11):1217. doi: 10.3390/vaccines9111217 (PMC8623218; doi:10.3390/vaccines9111217)
Supplement: Supplementary file 1 [file vaccines-09-01217-s001.zip › vaccines-1396578-supplementary.pdf]

Table S1. List of the chronic or onco-haematological diseases considered in the eligibility criteria.

Chronic heart disease;
Conditions at increased risk of aspiration of secretions (e.g. cerebral palsy, neuromuscular diseases, other causes of severe disability);
Adrenal cortical insufficiency;
Severe chronic kidney disease (e.g. nephrotic syndrome*, dialysis ...);
Chronic liver disease;
Diabetes mellitus;
Haemophilia and other coagulation disorders;
Anemia and haemoglobinopathy;
Severe obesity;
Chronic inflammatory diseases and malabsorption syndromes;
Patients with Cochlear implant;
Patients with cerebrospinal fluid fistula;
Chronic salicylate therapy;
Patient waiting for major surgery;
Primary or secondary immunodeficiencies (due to immunosuppressive therapies, onchohaematological diseaese, HIV infection, hematopoietic stem-cell transplantation);
Solid organ transplantation;
Surgical or functional asplenia.

* medical condition included only in the immunization schedule of Tuscany region

Table S2. Total number of surveys collected by medical condition.

Medical condition	Number of surveys collected
Chronic respiratory or lung disease	20 (15.8%)
Chronic heart disease	6 (4.7%)
Conditions at increased risk of aspiration of secretions	13 (10.3%)
Adrenal cortical insufficiency	4 (3.1%)
Severe chronic kidney disease	4 (3.1%)
Chronic liver disease	1 (0.8%)
Diabetes mellitus	13 (10.3%)
Hemophilia and other coagulation disorders	4 (3.1%)
Anemia and hemoglobinopathy	12 (9.5%)
Severe obesity	3 (2.4%)
Chronic inflammatory diseases and malabsorption syndromes	2 (1.6%)
Cochlear implant	6 (4.7%)
Cerebrospinal fluid fistula	1 (0.8%)
Chronic salicylate therapy	0 (0.0%)
Patient waiting for major surgery	2 (1.6%)
Primary or secondary immunodeficiencies	28 (22.0%)
Solid organ transplantation	1 (0.8%)
Surgical or functional asplenia	7 (5.5%)
